# Social inclusion: a fundamental PROM for evaluating recovery-oriented global mental health programmes

**DOI:** 10.1192/j.eurpsy.2023.131

**Published:** 2023-07-19

**Authors:** B. Puschner

**Affiliations:** Department of Psychiatry II, Ulm University, Günzburg, Germany

## Abstract

**Abstract:**

Social inclusion is a multidimensional concept, referring to ample participation in key social, cultural, economic and political activities. Since the turn of the century, social inclusion has become a guiding principle in policy recommendations of many international (United Nations, WHO, EU) and national bodies, aiming to improve the lives of people with mental ill-health. More recently, social inclusion has been increasingly used as an outcome measure, to test the effectiveness of complex interventions, especially in the field of global mental health.

This presentation will focus on: (1) current definitions and controversies in research on social inclusion for and with people with mental ill-health; (2) measuring social inclusion; and (3) the evidence-base of interventions to improve social inclusion. Special attention will be given to recovery-oriented interventions in global mental health such as peer support.

In summary, over the last years, impressive conceptual and methodological advances have been made to transform policy rhetoric into meaningful and effective interventions. However, challenges remain, including consensus on culturally appropriate measurement of social inclusion, and balancing the roles and responsibilities of all stakeholders (service users, mental health service providers, wider society) across the entire exclusion-inclusion continuum to promoting social inclusion and mental health.

**Disclosure of Interest:**

None Declared

